# Mesh–fixation combinations and chronic postoperative inguinal pain after laparoscopic groin hernia repair: nationwide cohort study

**DOI:** 10.1093/bjsopen/zrag073

**Published:** 2026-07-04

**Authors:** Bengt Novik

**Affiliations:** Department of Clinical Sciences, Danderyd Hospital, Karolinska Institute, Stockholm, Sweden

**Keywords:** chronic postsurgical pain, hernia mesh, mesh fixation, registry study, patient-reported outcomes, Swedish Hernia Register

## Abstract

**Background:**

Despite lower prevalence after laparoscopic approach, chronic postoperative inguinal pain (CPIP) remains a major concern. Large-scale comparisons of various mesh–fixation combinations with respect to CPIP risk are scarce. This study evaluated the association between commonly used mesh–fixation combinations and CPIP after laparoscopic groin hernia repair.

**Methods:**

This comparative cohort study analysed prospectively collected data from the nationwide Swedish Hernia Registry, including virtually all adult patients undergoing unilateral laparoscopic groin hernia repair in Sweden over a 6-year 4-month period. One year after surgery, a patient-reported outcomes survey assessed CPIP, defined as pain in the operated groin during the preceding week that affected daily activities. This study also classified all patients who underwent reoperation within the first 15 months as CPIP-positive respondents. Multivariable logistic regression estimated adjusted odds ratios for mesh–fixation combinations recorded in > 100 procedures.

**Results:**

Of 15 360 eligible patients, 10 525 (68.5%) responded (mean age 59 years; 32.0% women). Three mesh types (flat heavyweight, flat lightweight, and ‘three-dimensional’) and five fixation modes (absorbable tacks, metal tacks, fibrin glue, self-gripping micro-hooks, and non-fixation) yielded 12 analyzable combinations. The lowest odds of CPIP were observed with three options: heavyweight flat mesh without fixation (reference category; adjusted odds ratio 1.0), lightweight mesh with fibrin glue (adjusted odds ratio 1.0, 95% confidence interval 0.75 to 1.4), and lightweight mesh with self-gripping micro-hooks (adjusted odds ratio 0.94, 95% confidence interval 0.61 to 1.4).

**Conclusion:**

Heavyweight flat mesh without fixation was associated with one of the lowest odds of CPIP, while representing the simplest and least costly alternative. Together with previous registry evidence indicating low recurrence risk, these findings support considering this option as a pragmatic default strategy in laparoscopic groin hernia repair.

## Teaser

Chronic postoperative inguinal pain (CPIP) is reduced after laparoscopic groin hernia repair, yet remains a major concern. A nationwide Swedish Hernia Registry analysis compared common mesh–fixation combinations in unilateral laparoscopic repair. Heavyweight flat mesh without fixation—the simplest and least expensive option—showed among the lowest CPIP odds.

## Introduction

Chronic postoperative inguinal pain (CPIP) is the principal long-term adverse outcome after groin (inguinal and femoral) hernia repair^1^. It is commonly defined as local pain persisting beyond a normal healing period of no more than 3–6 months^2^.

Laparoscopic—including robotic-assisted—transabdominal preperitoneal (TAPP) and totally extraperitoneal (TEP) repairs have been associated with lower CPIP risk than open procedures^2–5^. This advantage has helped drive the steady increase of minimally invasive repairs over the past three decades. In 2023, an estimated 27% of groin hernia repairs in high-income and upper-middle-income countries were performed laparoscopically^6^. Nonetheless, a substantial proportion of patients still experience CPIP after minimally invasive repair^7^.

Further reductions in CPIP risk may depend on selecting an optimal combination of mesh type and fixation mode. Lightweight mesh (LWM), anatomically contoured three-dimensional (3D) mesh, absorbable tacks, adhesives, and self-fixating devices (most commonly Progrip^®^, Medtronic, Dublin, Ireland) have been introduced with the aim of reducing pain and recurrence^8–12^. However, support for these technologies often rests on theoretical rationale, pre-clinical experiments, and underpowered patient trials. Large-scale, producer-independent evaluations remain scarce^13–15^. Despite decades of controversy, no trial or meta-analysis has conclusively identified a clearly superior mesh–fixation combination. National registry data provide a unique opportunity to evaluate such combinations at scale and in routine clinical practice.

Most existing studies^16–20^ have examined mesh or fixation separately, which obscures potential interactions, increases confounding risk, and may yield misleading conclusions^21^. This limitation is reflected in the international HerniaSurge guidelines^2^, which address mesh and fixation in separate chapters. The recent update of the *Meshes* chapter^4^—based on evidence available through 2021—concludes that LWM in laparoscopic repair ‘*does not reduce the risk of postoperative pain but increases risk of recurrence.*’ However, several large-scale cohort studies^19–24^ published subsequently have reported findings that challenge these claims. These findings suggest that the issue remains unresolved and many laparoscopic hernia surgeons still favour LWM.

An important issue is the scarcity of sizable studies assessing mesh and fixation as a *combined* exposure. Recently, some studies^21,24^ have been published but none of them had CPIP as the primary outcome. Using data from the Swedish Hernia Registry (SHR), the 2022 report^21^ was the first large-scale analysis of multiple mesh–fixation pairings in relation to reoperation risk after laparoscopic repair. The findings indicated that mesh type and fixation mode interact and therefore should be analysed jointly. Notably, heavyweight polypropylene mesh without fixation—the least costly option—was among the combinations associated with the lowest reoperation risk. The LWM categories were generally associated with higher reoperation rates, consistent with the HerniaSurge guidelines conclusions. However, one important exception was LWM fastened with fibrin glue, providing wide non-penetrating fixation^11^. Other LWM combinations involving broad, atraumatic fixation—for example Progrip^®^—were not included in that study but appear effective in more recent reports^19,20,22–24^, including in laparoscopic repair. Experimental work suggests that so-called heavyweight mesh (HWM), owing to its greater stiffness, may not require fixation even in large defects, but such studies did not evaluate CPIP^25^.

Justifying the higher cost of certain options requires evidence that these combinations reduce CPIP and recurrence risks compared with cheaper alternatives.

The objective of this study was to evaluate the association between commonly used mesh–fixation combinations in laparoscopic repair and their relative odds of CPIP in real-world practice. The primary hypothesis was that LWM combined with non-penetrating fixation—either glue or self-fixation technology—would be associated with reduced risk of CPIP.

## Methods

The study is reported in accordance with the STROBE guidelines for observational studies^26^ and the RECORD extension for routinely collected clinical data^27^. Ethical approval was obtained from the Regional Ethical Review Board in Stockholm (EPN 2008/1082-31/2, amendments EPN 2014/2176-32 and EPN 2018/2050-32). Registration identifier: NCT04838028.

### Study design and setting

This nationwide cohort study included all patients aged ≥ 15 years who underwent unilateral laparoscopic groin hernia repair and were recorded in the SHR during a 6-year 4-month period. The SHR is publicly funded, independent of commercial interests, and captures virtually all adult groin hernia repairs in Sweden^28^. An English summary of its data acquisition, management, lifelong follow-up, and validation procedures is available on the SHR website^29^.

### Cohort

**Fig. 1:**
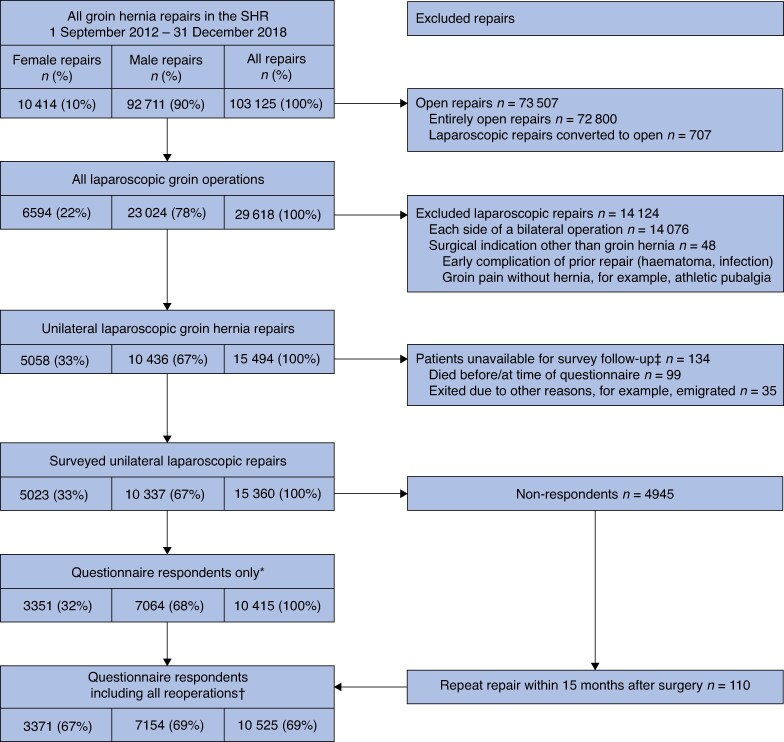
Flowchart In the SHR, each repaired groin is recorded as 1 post; a bilateral repair renders 2 posts. A reoperated hernia means a repeat repair in the same groin, irrespective of anatomical types of hernia. In the bottom row of the left column, percentages refer to respondents within each category. Adapted from Novik et al^7^. SHR, Swedish Hernia Registry. *Respondents according to the alternative CPIP definition. †Respondents according to the primary CPIP definition.

A prospective patient-reported outcomes project within the SHR included all groin hernia repairs performed between 1 September 2012 and 31 December 2018. One year after surgery, all surviving patients were invited to complete a self-administered four-item questionnaire^30^; the first item assessed current pain. For the present analysis, all unilateral laparoscopic repairs from this project were included (*Fig. 1*), using a data set linking patient-reported outcomes and reoperations to 6 November 2020. Bilateral repairs were excluded because the questionnaire provided one response per patient and could not differentiate symptoms by side^7^.

### Endpoints

#### Primary outcome

The prevalence of CPIP was surveyed 1 year after surgery. Patients rated their highest degree of groin pain during the preceding week, using the first item of the validated Short-Form Inguinal Pain Questionnaire^30,31^. In the present study, responses on the 7-point Likert scale were dichotomized as CPIP-positive or CPIP-negative. CPIP was defined as pain that *could not be ignored, and interfered with concentration on chores/activities* (*Table 1*).

**Table 1:** Chronic pain item in the SHR survey

*Rate the worst level of pain you have felt in the operated groin during the recent week:*	
1.	No pain
2.	Pain, could easily be ignored
3.	Pain, could not be ignored, but did not affect daily activities
4.	Pain, could not be ignored and interfered with concentration on chores/activities
5.	Pain, prevented most activities
6.	Pain, required bed rest
7.	Pain, prompted seeking immediate medical help

Patient-reported CPIP at 1 year after surgery. In this study, levels 4–7 were regarded as CPIP. SHR, Swedish Hernia Registry. CPIP, chronic postoperative inguinal pain.

A limitation of the questionnaire was its inability to attribute symptoms accurately to the index repair in patients who had undergone reoperation before the survey. Pain is a common symptom of recurrence and meets the time-based definition of CPIP if present beyond 3–6 months^1^. To avoid underestimation of CPIP prevalence, patients who underwent repeat repair in the same groin within 15 months after surgery were classified as CPIP-positive, regardless of survey response. Under this broader definition, initial non-respondents who had undergone a new repair were re-classified as ‘respondents’ (*Fig. 1*).

#### Alternative outcome

A slightly narrower CPIP definition was applied for sensitivity analysis. This outcome did not account for any repeat repairs before the survey. The questionnaire responses were analysed as reported, and no initial non-respondents were included (*Fig. 1*). This definition aligns with some previous SHR studies^30,32–34^.

### Variables

#### Exposures

The exposures of interest were type of mesh and mode of fixation, categorized according to a standardized SHR classification launched on 1 January 2012. To cover both established and emerging devices, it groups products by generic properties. *Tables 2,3* present an edited version. An 8-month familiarization period preceded the study window, which minimized the risk of misclassification.

**Table 2:** Mesh classification adapted from the Swedish Hernia Registry form

	Material	Shape	Weight/m^2^	Examples of current brand names
Heavyweight mesh	PP PET	Flat sheet	≥ 50 g	PP: BBraun Premilene, *and* Optilene; DynaMesh PP; Medtronic Surgipro, *and* Parietene; BD Bard Mesh; Ethicon Prolene PET: Medtronic Parietex, *and* Versatex; Ethicon Mersilene
Lightweight mesh	Single or composite plastic. Main polymer base: PP, PET, or PVDF	Flat sheet	< 50 g	BBraun Optilene LP; DynaMesh PP Light; BD Bard Soft Mesh; Ethicon Ultrapro, *and* Prolene Soft; Medtronic Parietene Macroporous, *and* all Progrip mesh variants
3D mesh	Anatomically preshaped, any material	Curved	any	Cousin 4DMesh; Medtronic Dextile; BD 3D Max; DynaMesh Endolap 3D
*Other* *	PVDF			DynaMesh products only
	PTFE	expanded (ePTFE) condensed (cPTFE)		Gore Dualmesh BBraun Omyra
	Absorbable synthetic = ‘biosynthetic’			TIGR Matrix; Gore BioA; BD Phasix
	Biologic			Cook Biodesign; Allergan Strattice, *and* AlloDerm

*Constitutes several categories in the original SHR classification, but here, the ones less frequently used in laparoscopic groin hernia repair are merged into the *Other* category. PP, polypropylene; PET, polyester; PVDF, polyvinylidene fluoride; PTFE, polytetrafluoroethylene.

**Table 3:** Fixation classification adapted from the Swedish Hernia Registry form

	Material	Examples of current brand names
No fixation	N/A	N/A
Absorbable tacks	For example, poly glycolide-co-lactide, *or* polydioxanone and L-lactide/glycolide copolymer	Ethicon SecureStrap; Medtronic AbsorbaTack; BD SorbaFix
Metal tacks or staples	Titanium alloy	Medtronic Protack, *and* EndoHernia; BD CapSure
Fibrin glue	Fibrinogen + thrombin (extracted from human blood donors)	Baxter Tisseel; Ethicon Evicel
Synthetic glue	Predominantly cyanoacrylates	GEM Glubran; BBraun Histoacryl; Ethicon Dermabond
Progrip^®^	Absorbable Velcro-type microhooks, only available in some PP or PET lightweight meshes from Medtronic	Medtronic mesh brands: ProGrip, Parietene ProGrip, Parietex ProGrip, ProGrip Laparoscopic
*Other* *	Sutures, permanent plastic tacks, pre-glued mesh, etc.	BD PermaFix plastic tacks, *and* Adhesix pre-glued mesh

*Constitutes several categories in the original SHR classification, but here, the ones less frequently used in laparoscopic groin hernia repair are merged into the *Other* category.

Mesh type and fixation mode were combined into a single exposure variable that initially included all mesh–fixation categories with more than 100 observations.

#### Covariates

Nine covariates were included in the multivariable model: sex, age, body mass index (BMI), American Society of Anesthesiologists (ASA) grade, recurrent *versus* primary repair, hernia anatomy, hernia defect size, repair method (TEP *versus* TAPP), and surgical centre. They were selected based on a prespecified screening analysis of the same cohort that evaluated 18 preoperative and intraoperative candidate risk factors^7^. Variables not meaningfully improving model fit (*P* ≥ 0.10 in the screening analysis) were omitted from the final model: smoking, diabetes, collagen disease, chronic obstructive pulmonary disease, bleeding diathesis, immunosuppression, side, emergency status, and surgeon level (consultant *versus* resident).

### Data access and cleaning

The raw SHR data set was manually reviewed to identify implausible or inconsistent entries. Values were corrected only when the intended entry could be inferred with high confidence; otherwise, data were recoded as missing. In several cases, uncertainties were resolved by contacting the surgical centre.

### Statistical methods

A simulated power analysis assumed an overall CPIP prevalence of 15% (based on previous SHR studies)^30,34^, a low-risk reference (odds ratio (OR) 1.0) category, and most mesh–fixation combinations clustering near OR 1.2. The model simulated mesh–fixation category sizes of 50–1000 to estimate their power to detect significantly true ORs of, respectively, 1.1, 1.3, and 1.5, the latter of which was prespecified as clinically important. These calculations were performed in R, version 4.5.2 (R Foundation for Statistical Computing, Vienna, Austria, http://www.r-project.org/).

Associations between CPIP and sufficiently large mesh–fixation groups, as well as co-variates, were assessed using multivariable logistic regression. For each variable category, an adjusted OR (aOR) and 95% confidence interval (c.i.) were estimated. Statistical significance was defined as *P* < 0.050 (2-tailed). Variables with missing data were assigned a subcategory ‘Missing data’, so that affected patients could still be included in the analysis. These calculations were performed using IBM SPSS Statistics^®^, version 28.0.1.1 (IBM, Armonk, New York, USA).

## Results

### Participants and CPIP prevalence

During the study period, approximately 16 000 groin hernia repairs were performed annually in Sweden, whereas the laparoscopic proportion increased from 8% in 2012 to 36% in 2018.

A detailed cohort description has been published (open access)^7^. In brief, among 15 360 patients who underwent unilateral laparoscopic repair at 82 centres, 10 525 (68.5%) were respondents, including 110 initial non-respondents who underwent reoperation (*Fig. 1*). The men-to-women ratio of approximately 2:1 reflects the higher use of laparoscopy among women^2^. Respondents had a mean(standard deviation (s.d.), range) age of 59(15, 15–94) years, and their mean(s.d., range) BMI was 25(3.2, 15–55) kg/m^2^. Most of them were low risk (ASA I–II), whereas 761 (7.2%) were classified as ASA III–IV. Emergency surgery accounted for 238 (2.3%) repairs. Procedures comprised 8864 (84.2%) TEP repairs, 1589 (15.1%, including 39 robotically) TAPP repairs, and 72 (0.7%) minimally invasive repairs classified as unspecified or other.

Using the primary definition, CPIP was identified in 1827 patients (17.4%), whereas 1614 patients (15.5%) met the narrower, alternative definition.

### Variables

The most common mesh–fixation combinations comprised four types of mesh (flat heavyweight polypropylene or polyester mesh, LWM, and 3D mesh), and five modes of fixation (absorbable and metal tacks, fibrin glue, Progrip^®^, and no fixation).

**Fig. 2:**
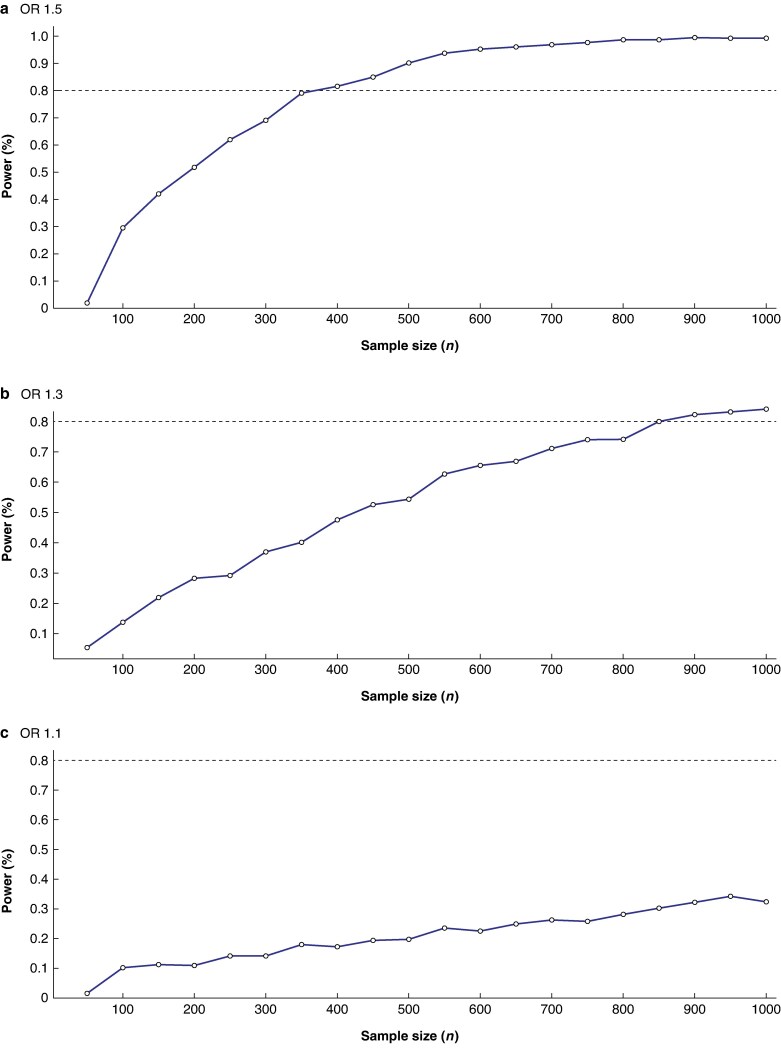
Simulated power analysis **a** OR 1.5, **b** OR 1.3, **c** OR 1.1. The curves demonstrate the power (Y axis) achieved by the sample size (X axis) per mesh–fixation combination. Each panel depicts the power to avoid a type II error of a determined, true OR level. The horizontal dashed line indicates 80% power. Assumptions: An overall CPIP prevalence of 15%; a low-risk reference (OR 1) category; most combinations cluster around OR 1.2. OR, odds ratio.

The simulated power analysis suggested that each mesh–fixation category should include approximately 350 repairs to achieve 80% power to detect the prespecified clinically important OR of 1.5 (*Fig. 2*). Hence, the initial lower limit of > 100 resulted in inclusion of several underpowered categories. In a preliminary analysis, heavyweight polypropylene *versus* polyester flat mesh—when having identical modes of fixation—demonstrated comparable CPIP estimates. To improve power, these two mesh types were merged and designated HWM. This generated four better-sized mesh–fixation categories, which together with eight LWM and 3D mesh options resulted in 12 categories accounting for 10 140 (96.3%) procedures, and were selected for the main analysis (*Table 4*). Remaining less frequent variants, along with repairs with missing mesh or fixation data, were merged into an *Uncategorized* group (n = 385), detailed in *Table S1*.

**Table 4:** Crude and multivariable logistic regression of CPIP at 1 year, by mesh–fixation combination

	Respondents	CPIP	Unadjusted		Adjusted	
	*n* (%)	*n* (%)*	OR	*P*†	aOR	*P*†
All mesh–combinations	10 525 (100%)	1827 (17.4%)				
**Heavyweight mesh**	1221 (11.6%)	248 (20.3%)				
No fixation	371 (3.5%)	58 (15.6%)	1.0 (reference)		1.0 (reference)	
Tacks, absorbable	351 (3.3%)	80 (22.8%)	1.6 (1.1, 2.3)	0.015	1.7 (1.1, 2.4)	0.012
Tacks, metal	345 (3.3%)	77 (22.3%)	1.6 (1.1, 2.3)	0.023	1.6 (1.1, 2.4)	0.016
Fibrin glue	154 (1.5%)	33 (21.4%)	1.5 (0.91, 2.4)	0.112	1.5 (0.93, 2.4)	0.098
**Lightweight mesh**	4988 (47.4%)	857 (17.2%)				
No fixation	1574 (15.0%)	304 (19.3%)	1.3 (0.95, 1.8)	0.102	1.3 (0.98, 1.8)	0.071
Tacks, absorbable	1006 (10.0%)	187 (18.6%)	1.2 (0.89, 1.7)	0.201	1.3 (0.95, 1.9)	0.093
Tacks, metal	168 (1.6%)	31 (18.5%)	1.2 (0.76, 2.0)	0.415	1.2 (0.76, 2.0)	0.378
Fibrin glue	1925 (18.3%)	290 (15.1%)	0.96 (0.70, 1.3)	0.780	1.0 (0.75, 1.4)	0.895
Progrip	315 (3.0%)	45 (14.3%)	0.90 (0.59, 1.4)	0.623	0.94 (0.61, 1.4)	0.772
**3D mesh**	3931 (37.3%)	671 (17.1%)				
No fixation	2992 (28.4%)	488 (16.3%)	1.1 (0.78, 1.4)	0.739	1.1 (0.81, 1.5)	0.521
Tacks, absorbable	745 (7.1%)	148 (19.9%)	1.3 (0.96, 1.9)	0.087	1.4 (=1.01, 2.0)	0.046
Fibrin glue	194 (1.8%)	35 (18.0%)	1.2 (0.75, 1.9)	0.464	1.1 (0.72, 1.8)	0.561
**Uncategorized combinations**						
Rare or unspecified	385 (3.7%)					

ORs and aORs are shown with 95% confidence intervals in parentheses. In the multivariable analysis, the outcomes are adjusted for the effect modifiers in *Table 5*. CPIP, chronic postoperative inguinal pain. OR, CPIP odds ratio; aOR, adjusted odds ratio of CPIP risk; 3D, three-dimensional. *Number of respondents with CPIP divided by total number of respondents within each mesh–fixation category; †Statistical significance was defined as *P* < 0.05 (2-tailed).

The demographic and clinical covariates and their impact on the multivariable model are shown in *Table 5*.

**Table 5:** The modifying effects of demographic and clinical covariates on CPIP in *Table 4*, multivariable logistic regression

	Patients *n* (%)	aOR	*P*
**All patients**	10 525 (100%)		
**Sex**			
Male	7154 (68.0%)	1.0 (reference)	
Female	3371 (32.0%)	1.0 (0.89, 1.2)	0.795
**Age (years)**			
15–30	491 (4.7%)	1.8 (1.4, 2.4)	< 0.001
30–45	1521 (14.5%)	1.2 (0.99, 1.5)	0.067
45–55	1840 (17.5%)	1.2 (0.97, 1.4)	0.092
55–65	2437 (23.2%)	1.2 (0.97, 1.4)	0.093
65–70	1462 (13.9%)	1.0 (reference)	
70–75	1362 (12.9%)	1.1 (0.90, 1.4)	0.324
>75	1412 (13.4%)	1.0 (0.80, 1.2)	0.865
**Body mass index (kg/m^2^)**			
<20	500 (4.8%)	1.3 (1.03, 1.7)	0.029
20–25	5255 (49.9%)	1.0 (reference)	
25–30	3757 (35.7%)	1.3 (1.2, 1.5)	< 0.001
>30	574 (5.5%)	1.6 (1.3, 2.0)	< 0.001
Missing or improbable value	439 (4.2%)		
**ASA grade**			
ASA 1	5498 (52.2%)	1.0 (reference)	
ASA 2	4266 (40.5%)	1.2 (1.1, 1.4)	0.002
ASA 3–4	761 (7.2%)	1.6 (1.3, 2.0)	< 0.001
**Primary/recurrent repair**			
Primary (first-time) repair	8122 (77.2%)	1.0 (reference)	
Recurrent repair	2403 (22.8%)	1.3 (1.1, 1.4)	< 0.001
**Hernia anatomy**			
Medial, Lateral, Combined Medial + Lateral	9451 (89.8%)	1.0 (reference)	
Femoral	676 (6.4%)	1.2 (0.95, 1.5)	0.131
Combined Femoral + Other	327 (3.1%)	1.3 (0.99, 1.7)	0.055
Other or missing value	71 (0.7%)		
**Hernia defect** Ø‡			
EHS I (< 1.5 cm)	2783 (26.4%)	1.2 (1.1, 1.4)	0.001
EHS II–III (≥ 1.5 cm)	7734 (73.5%)	1.0 (reference)	
Missing value	8 (0.1%)		
**Repair method**			
TEP	8864 (84.2%)	1.0 (reference)	
TAPP	1589 (15.1%)	0.93 (0.79, 1.1)	0.426
MIS, other	72 (0.7%)		
**Surgical centre**†	10 525 (100%)	1.0 (0.997, 1.005)	0.608

Values are *n* (%) unless otherwise stated. aORs are shown with 95% confidence intervals in parentheses. Multivariable logistic regression, also adjusted for mesh–fixation combinations (*Table 4*). aOR, adjusted odds ratio of chronic postoperative inguinal pain risk; ASA, American Society of Anesthesiologists; EHS, European Hernia Society classification; TEP, totally extraperitoneal; TAPP, transabdominal preperitoneal; MIS, minimally invasive surgery. †Whereas the mean OR = 1.0, the ORs of each of the 82 contributing centres differed significantly; ‡defect Ø: EHS II, 1.5–3 cm; EHS III, > 3 cm.

### Main analysis

The average overall CPIP prevalence was 17.4%, ranging from 14.3% to 22.8% across mesh–fixation combinations (*Table 4*). HWM without fixation served as reference category, and had a CPIP prevalence of 15.6%, lower than the mean. In the multivariable main analysis, three combinations were associated with significantly increased odds of CPIP: HWM with absorbable or metal tacks, and 3D mesh with absorbable tacks. Other combinations yielding numerically higher CPIP prevalences did not reach statistical significance. The unadjusted analysis yielded comparable results, indicating that the co-variates were rather evenly distributed among the mesh–fixation categories.

### Sensitivity analysis

The sensitivity analysis (*Table S2*) repeated the main analysis using the alternative, narrower CPIP definition, which was based solely on questionnaire responses and did not account for repeat repairs during follow-up. Under this definition, CPIP was identified in 1614 (15.5%) of 10 415 respondents. The overall pattern of results closely mirrored the main analysis, although now only one mesh–fixation combination—HWM with metal tacks (aOR 1.7, 95% c.i. 1.1 to 2.5)—remained significantly differing from the reference.

## Discussion

This nationwide registry-based study provides large-scale evaluation of multiple mesh–fixation combinations in relation to CPIP risk. With a 69% 1-year response rate, 10 525 of 15 360 eligible patients contributed to the main analysis.

Three main findings emerged. First, HWM without fixation (simplest and least expensive option) was among the combinations with the lowest CPIP odds. Secondly, fibrin glue and Progrip®, in contrast to tacks or no fixation, seemed to mitigate the increased CPIP risk otherwise observed for LWM. Third, tack fixation was associated with higher CPIP risk, significantly in three categories, with similar directional trends in the remaining two.

Although data collection ended some years ago, the assessed generic mesh–fixation categories reflect contemporary practice, and the fundamental principles of minimally invasive groin hernia repair have not changed^35^.

A previous SHR study^21^ on recurrence risk demonstrated that mesh and fixation interact, and that analysing either variable in isolation may be misleading. This interdependence likely contributes to inconsistent findings in earlier studies^16–19,36^ that examined only one component. Randomized clinical trials (RCTs) can address this by holding the counterpart constant, for example, using identical fixation when comparing two meshes, or vice versa^37^. However, such designs inevitably limit the range of combinations tested and reduce generalizability. An RCT evaluating multiple mesh–fixation pairings simultaneously would be challenging to conduct at scale. In contrast, sufficiently large and detailed registry data enable concurrent analysis of numerous real-world combinations^2,38^. Valid inference requires careful confounder control. In the preparatory study, all available preoperative and intraoperative SHR variables (except mesh and fixation) were screened to identify potential effect modifiers. This provided an extensive yet statistically appropriate set of co-variates in the present model^7^.

This study was designed to estimate relative odds across mesh–fixation combinations, rather than absolute CPIP prevalence. A previous analysis of the SHR survey project, including open repairs, indicated a substantially lower CPIP prevalence among non-respondents^21,30^.

Severe tack-related pain is a recognized complication and can be debilitating^11^. When tissue-penetrating fixation is applied judiciously within anatomically safe zones, the risk of serious injury is very low^17,35^. Nevertheless, neither absorbable nor metal tacks conferred any advantage regarding CPIP in the present analysis or regarding recurrence in our previous SHR study^21^. In the absence of demonstrable benefit, and given the additional cost, tacking may be difficult to justify outside selected bailout situations.

Fibrin glue was associated with lower CPIP risk only with LWM. This observation aligns with empirical experience: effective fibrin fixation requires close mesh–tissue contact during polymerization, which is readily achieved with a pliable LWM but difficult with more rigid meshes^21^. Whether use of fibrin glue with meshes other than LWM reflects surgeon expertise, and consequently the results, is speculative.

The strengths of this study are using data from the SHR, which is publicly funded, prospectively collected, validated, and free of commercial influence, enabling robust multivariable analyses^38,39^.

In this study, all eligible unilateral laparoscopic repairs were included consecutively and unselected, yielding broad representation of surgeons, institutions, and patients, which enhances generalizability^40^. Follow-up was conducted independently of operating units and industry, minimizing observer bias.

Because CPIP prevalence and severity are time-dependent^41^, the uniform 1-year follow-up strengthens validity and comparability. Information bias was reduced through thorough data review before analysis. The number of included relevant co-variates and level of multivariable adjustment exceeded those of most previous reports.

The 69% follow-up rate is robust in a study involving all operated individuals, including those limited by cognitive, physical, linguistic, or logistical barriers to questionnaire completion. Comparable CPIP registry studies^42–44^ have reported response rates of 39%, 63%, and 65%, respectively.

Finally, although the SHR survey has provided data to earlier publications^30,32,33,45^, this study and the preparatory analysis are the first to include the entire project period, thereby achieving its maximal statistical power^7^.

However, there are limitations. The simulated power analysis suggested that most analysed categories were sufficiently large to detect associations corresponding to an OR of 1.5 or greater. Smaller absolute risk differences, for example, corresponding to 1–2 additional cases of CPIP per 100 patients, would require substantially larger sample sizes to demonstrate statistically. Consequently, the absence of statistical significance for some comparisons should be interpreted cautiously, and these estimates may be considered hypothesis-generating.

Although adjustment included key clinical and centre-level variables, residual confounding related to unmeasured patient factors and surgeon-level decision-making cannot be excluded in observational studies^39^. The SHR does not record geographic residence, race, socioeconomic status, preoperative pain characteristics, or psychiatric history. Within the Swedish universal healthcare system, however, these factors are less likely to affect materially access to care or systematically bias hernia implant selection.

The surgeon’s rationale for choosing laparoscopic repair or a specific mesh–fixation combination was not recorded. Mesh classification did not distinguish lightweight from standard variants within 3D meshes. The registry did not capture mesh size, pore characteristics, fixation sites, number of tacks, or volume of glue. The repairs with suture or synthetic glue fixation were too few for meaningful analysis. Although mesh size could influence fixation requirements, the recommendation of a minimum 10 × 15 cm mesh has long been standard practice in Sweden^35^. Large- and small-pore variants were not distinguished. However, a recent Herniamed registry study^44^ found no differences in groin pain on exertion or requiring treatment at 1 year.

Biologic and biosynthetic implants were too few for separate analysis. Recent reports—a cohort study^46^ of a biosynthetic mesh (Phasix®, BD, Franklin Lakes, NJ, USA), and a RCT^47^ of biologic patches—found neither superior to conventional synthetic alternatives.

In laparoscopic repair, fascial defects are customarily left open and bridged with a mesh^35^, consistent with Swedish practice during the study period. More recently, some have proposed closure of medial defects to reduce seroma and recurrence risks, and to simplify or obviate fixation^48^. Others have raised cautions, in particular regarding risk of nerve injury, and currently evidence remains insufficient^49^. The present study could not address this issue.

Some degree of patient-reported misclassification of CPIP is inevitable^50^, but is presumably non-differential across mesh–fixation groups and unlikely to materially bias ORs.

Finally, the findings apply specifically to laparoscopic repairs, and cannot be extrapolated to open mesh repairs, such as the Lichtenstein procedure.

In conclusion, different mesh–fixation combinations were associated with differing odds of CPIP in this nationwide cohort. The favourable outcomes observed with HWM without fixation, despite being the simplest and most economical option, support its consideration as a pragmatic default strategy. Non-penetrating fixation (glue or Progrip^®^) appeared to reduce the CPIP risk observed with LWMs. However, a RCT comparing LWM with fibrin glue or Progrip^®^, or non-fixated 3D mesh, with HWM without fixation would require several thousand participants per arm to detect a reduction of 1–2 cases of CPIP per 100 patients. This may partly explain the limited randomized evidence available.

## Supplementary Material

zrag073_Supplementary_Data

## Data Availability

The data set analysed in the present study belongs to the Swedish Hernia Registry and cannot be provided by the author. To access the data, a separate application must be made to the Registry.
