# Supplementary material for: Mesh–fixation combinations and chronic postoperative inguinal pain after laparoscopic groin hernia repair: nationwide cohort study
Source: BJS Open. 2026 Jul 4;10(4):zrag073. doi: 10.1093/bjsopen/zrag073 (PMC13332403; doi:10.1093/bjsopen/zrag073)
Supplement: zrag073_Supplementary_Data [file zrag073_supplementary_data.docx]

Mesh–fixation combinations and chronic postoperative inguinal pain after laparoscopic groin hernia repair: nationwide cohort study

**Bengt Novik**

Department of Clinical Sciences, Danderyd Hospital, Karolinska Institute, Stockholm, Sweden

| Supplementary Figures and Tables |  |
| --- | --- |
| Table S1a. Uncategorized meshes & fixations, ordered by Mesh | *page 2* |
| Table S1b. Uncategorized meshes & fixations, ordered by Fixation | *page 4* |
| Table S2. Multivariable main analysis, with alternative CPIP definition | *page 6* |

# Table S1a. Components of the *Uncategorized* category in Table 4, ordered by Mesh

| **Mesh** | **Fixation** | **All** | | **Respondents** | |
| --- | --- | --- | --- | --- | --- |
|  |  | n | *%* | n | *%* |
|  |  |  |  |  |  |
| **StdPPM** | Sutures, permanent | 33 |  | 24 |  |
| *Standard* | Sutures, long-term absorbable | 6 |  | 3 |  |
| *polypropylene mesh* | Tacks, perm plastic | 3 |  | 2 |  |
|  | Glue, cyanoacrylate | 1 |  | 1 |  |
|  | Glue, other | 2 |  | 1 |  |
|  | Fixation: Yes, but unknown type | 1 |  | 1 |  |
|  | Fixation or not = Unknown | 1 |  | 1 |  |
|  | **Sum** | **47** | *8.4%* | **33** | *8.6%* |
|  |  |  |  |  |  |
| **LWM** | Sutures, permanent | 42 |  | 29 |  |
| *Lightweight mesh* | Sutures, long-term absorbable | 27 |  | 20 |  |
|  | Sutures, short-term absorbable | 1 |  | 1 |  |
|  | Tacks, permanent plastic | 4 |  | 4 |  |
|  | Glue, cyanoacrylate | 17 |  | 11 |  |
|  | Glue, other | 5 |  | 4 |  |
|  | Glue, unknown type | 1 |  | 1 |  |
|  | Adhesix mesh | 5 |  | 5 |  |
|  | Self-adhesive, other | 1 |  | 1 |  |
|  | Fixation: Yes, but unknown type | 2 |  | 1 |  |
|  | Fixation or not = Unknown | 3 |  | 3 |  |
|  | **Sum** | **108** | *19%* | **80** | *21%* |
|  |  |  |  |  |  |
| **3D mesh** | Sutures, permanent | 4 |  | 2 |  |
| *anatomically shaped* | Sutures, long-term absorbable | 5 |  | 4 |  |
|  | Sutures, short-term absorbable | 3 |  | 1 |  |
|  | Tacks, metal | 100 |  | 74 |  |
|  | Tacks, permanent plastic | 4 |  | 4 |  |
|  | Glue, cyanoacrylate | 15 |  | 9 |  |
|  | Glue, other | 1 |  | 0 |  |
|  | Fixation: Yes, but unknown type | 6 |  | 3 |  |
|  | Fixation or not = Unknown | 2 |  | 2 |  |
|  | **Sum** | **140** | *25%* | **99** | *26%* |
|  |  |  |  |  |  |
| **Polyester mesh** | Sutures, permanent | 4 |  | 0 |  |
|  | Sutures, long-term absorbable | 1 |  | 0 |  |
|  | Sutures, short-term absorbable | 1 |  | 1 |  |
|  | Tacks, permanent plastic | 2 |  | 1 |  |
|  | Glue, cyanoacrylate | 5 |  | 3 |  |
|  | Fixation: Yes, but unknown type | 1 |  | 1 |  |
|  | **Sum** | **14** | *3%* | **6** | *2%* |
|  |  |  |  |  |  |
| **PTFE** | Glue, fibrin | 2 |  | 1 |  |
|  | Glue, cyanoacrylate | 1 |  | 0 |  |
|  | **Sum** | **3** | *0.5%* | **1** | *0.3%* |

*(continued)*

*(continued)*

**Table S1a.** Components of the *Uncategorized* category in Table 4, ordered by Mesh

| **Mesh** | **Fixation** | **All** | | **Respondents** | |
| --- | --- | --- | --- | --- | --- |
|  |  | n | *%* | n | *%* |
|  |  |  |  |  |  |
| **Other synthetic** | Sutures, permanent | 2 |  | 0 |  |
| *composite mesh* | Sutures, long-term absorbable | 4 |  | 4 |  |
|  | Sutures, short-term absorbable | 2 |  | 2 |  |
|  | Tacks, absorbable | 2 |  | 2 |  |
|  | Tacks, permanent plastic | 3 |  | 2 |  |
|  | Glue, cyanoacrylate | 7 |  | 2 |  |
|  | Glue, other | 1 |  | 0 |  |
|  | Fixation: Yes, but unknown type | 1 |  | 1 |  |
|  | **Sum** | **22** | *3.9%* | **13** | *3.4%* |
|  |  |  |  |  |  |
| **Other synthetic** | No fixation | 61 |  | 39 |  |
| *non-absorbable* | Sutures, long-term absorbable | 3 |  | 2 |  |
|  | Tacks, metal | 11 |  | 6 |  |
|  | Tacks, absorbable | 72 |  | 48 |  |
|  | Tacks, permanent plastic | 1 |  | 0 |  |
|  | Glue, fibrin | 1 |  | 0 |  |
|  | Glue, cyanoacrylate | 4 |  | 2 |  |
|  | Fixation: Yes, but unknown type | 1 |  | 0 |  |
|  | **Sum** | **154** | *28%* | **97** | *25%* |
|  |  |  |  |  |  |
| **Biosynthetic mesh** | No fixation | 18 |  | 17 |  |
| *synthetic absorbable* | Tacks, metal | 1 |  | 1 |  |
|  | Tacks, absorbable | 3 |  | 3 |  |
|  | **Sum** | **22** | *3.9%* | **21** | *5.5%* |
|  |  |  |  |  |  |
| **Biologic patch** | No fixation | 6 |  | 4 |  |
|  | Tacks, absorbable | 2 |  | 1 |  |
|  | **Sum** | **8** | *1.4%* | **5** | *1.3%* |
|  |  |  |  |  |  |
| **Mesh** | No fixation | 14 |  | 8 |  |
| *uncategorized* | Sutures, permanent | 1 |  | 0 |  |
|  | Sutures, uncategorized | 1 |  | 1 |  |
|  | Tacks, metal | 2 |  | 2 |  |
|  | Tacks, absorbable | 1 |  | 1 |  |
|  | Glue, fibrin | 1 |  | 1 |  |
|  | Fixation: Yes, but unknown type | 8 |  | 7 |  |
|  | Fixation or not = Unknown | 11 |  | 9 |  |
|  | **Sum** | **39** | *7.0%* | **29** | *7.5%* |
|  |  |  |  |  |  |
| **MIS method, mesh, fixation:** All 3 variables uncategorized | | **1** | *0.2%* | **1** | *0.3%* |
|  |  |  |  |  |  |
| **Total sum** |  | **558** | *100%* | **385** | *100%* |
|  | Total sum = % of all | 15 360 | *3.6%* | 10 525 | *3.7%* |
|  |  |  |  |  |  |

# Table S1b. Components of the *Uncategorized* category in Table 4, ordered by Fixation

| **Fixation** | | **Mesh** | **All** |  | **Respondents** | |
| --- | --- | --- | --- | --- | --- | --- |
|  |  |  | n | *%* | n | *%* |
|  | |  |  |  |  |  |
| **No fixation** | | Other synthetic | 61 |  | 39 |  |
|  | | Biosynthetic | 18 |  | 17 |  |
|  | | Biologic | 6 |  | 4 |  |
|  | | Yes, but uncategorized | 14 |  | 8 |  |
|  | | **Sum** | **99** | *18%* | **68** | *18%* |
|  | |  |  |  |  |  |
| **Sutures** | | StdPPM | 33 |  | 24 |  |
| *permanent* | | LWM | 42 |  | 29 |  |
|  | | 3D | 4 |  | 2 |  |
|  | | Polyester | 4 |  | 0 |  |
|  | | Other composite synthetic | 2 |  | 0 |  |
|  | | Yes, but uncategorized | 1 |  | 0 |  |
|  | | **Sum** | **86** | *15%* | **55** | *14%* |
|  | |  |  |  |  |  |
| **Sutures** | | StdPPM | 6 |  | 3 |  |
| *long-term absorbable* | | LWM | 27 |  | 20 |  |
|  | | 3D | 5 |  | 4 |  |
|  | | Polyester | 1 |  | 0 |  |
|  | | Other composite synthetic | 4 |  | 4 |  |
|  | | Other synthetic | 3 |  | 2 |  |
|  | | **Sum** | **46** | *8.2%* | **33** | *8.6%* |
|  | |  |  |  |  |  |
| **Sutures** | | LWM | 1 |  | 1 |  |
| *short-term absorbable* | | 3D | 3 |  | 1 |  |
|  | | Polyester | 1 |  | 1 |  |
|  | | Other composite synthetic | 2 |  | 2 |  |
|  | | **Sum** | **7** | *1.3%* | **5** | *1.3%* |
|  | |  |  |  |  |  |
| **Sutures**, uncategorized | | Mesh, uncategorized | **1** | *0.2%* | **1** | *0.3%* |
|  | |  |  |  |  |  |
| **Tacks** | | 3D | 100 |  | 74 |  |
| *metal* | | Other composite synthetic | 11 |  | 6 |  |
|  | | Biosynthetic | 1 |  | 1 |  |
|  | | Yes, but uncategorized | 2 |  | 2 |  |
|  | | **Sum** | **114** | *20%* | **83** | *22%* |
|  | |  |  |  |  |  |
| **Tacks** | | Other composite synthetic | 2 |  | 2 |  |
| *absorbable* | | Other synthetic | 71 |  | 47 |  |
|  | | Biosynthetic | 3 |  | 3 |  |
|  | | Biologic | 2 |  | 1 |  |
|  | | Yes, but uncategorized | 2 |  | 2 |  |
|  | | **Sum** | **80** | *14%* | **55** | *14%* |
|  | |  |  |  |  |  |
| **Tacks** | StdPPM | | 3 |  | 2 |  |
| *plastic permanent* | LWM | | 4 |  | 4 |  |
|  | 3D | | 4 |  | 4 |  |
|  | Polyester | | 2 |  | 1 |  |
|  | Other composite synthetic | | 3 |  | 2 |  |
|  | Other synthetic | | 1 |  | 0 |  |
|  | **Sum** | | **17** | *3.0%* | **13** | *3.4%* |

*(continued)*

*(continued)*

**Table S1b.** Components of the *Uncategorized* category in Table 4, ordered by Fixation

| **Fixation** | **Mesh** | **All** | | **Respondents** | |
| --- | --- | --- | --- | --- | --- |
|  |  | n | *%* | n | *%* |
|  |  |  |  |  |  |
| **Glue** | PTFE | 2 |  | 1 |  |
| *fibrin* | Other synthetic | 1 |  | 0 |  |
|  | Yes, but uncategorized | 1 |  | 1 |  |
|  | **Sum** | **4** | *0.7%* | **2** | *0.5%* |
|  |  |  |  |  |  |
| **Glue** | StdPPM | 1 |  | 1 |  |
| *cyanoacrylate* | LWM | 17 |  | 11 |  |
|  | 3D | 15 |  | 9 |  |
|  | Polyester | 5 |  | 3 |  |
|  | PTFE | 1 |  | 0 |  |
|  | Other composite synthetic | 7 |  | 2 |  |
|  | Other synthetic | 4 |  | 2 |  |
|  | **Sum** | **50** | *9.0%* | **28** | *7.3%* |
|  |  |  |  |  |  |
| **Glue** | StdPPM | 2 |  | 1 |  |
| *other* | LWM | 5 |  | 4 |  |
|  | 3D | 1 |  | 0 |  |
|  | Other composite synthetic | 1 |  | 0 |  |
|  | **Sum** | **9** | *1.6%* | **5** | *1.3%* |
|  |  |  |  |  |  |
| **Glue,** *uncategorized* | LWM | **1** | *0.2%* | **1** | *0.3%* |
|  |  |  |  |  |  |
| **Adhesix**® | Preglued LWM | **5** | *0.9%* | **5** | *1.3%* |
|  |  |  |  |  |  |
| **Self-adhesive,** *other* | LWM | **1** | *0.2%* | **1** | *0.3%* |
|  |  |  |  |  |  |
| **Fixation** | StdPPM | 1 |  | 1 |  |
| *Yes, but unknown type* | LWM | 2 |  | 1 |  |
|  | 3D | 6 |  | 3 |  |
|  | Polyester | 1 |  | 1 |  |
|  | Other composite synthetic | 1 |  | 1 |  |
|  | Other synthetic | 1 |  | 0 |  |
|  | Yes, but uncategorized | 8 |  | 7 |  |
|  | **Sum** | **20** | *3.6%* | **14** | *3.6%* |
|  |  |  |  |  |  |
| **Fixation or not** | StdPPM | 1 |  | 1 |  |
| *Unknown* | LWM | 3 |  | 3 |  |
|  | 3D | 2 |  | 2 |  |
|  | Yes, but uncategorized | 11 |  | 9 |  |
|  | Unknown if mesh was used | 1 |  | 1 |  |
|  | **Sum** | **18** | *3.2%* | **16** | *4.2%* |
|  |  |  |  |  |  |
| **Total sum** |  | **558** | *100%* | **385** | *100%* |
|  | Total sum = % of all | 15 360 | *3.6%* | 10 525 | *3.7%* |
|  |  |  |  |  |  |

| **Table S2.** Multivariable main analysis, with alternative CPIP definition | | | | | | | |
| --- | --- | --- | --- | --- | --- | --- | --- |
|  | | | | | | | |
|  | **Respondents** | | **CPIP** | |  |  |  |
| **Variable** | **n** | (%) | **n** | (%) | **aOR** | *CI 95%* | ***P*** |
| **Mesh–Fixation** combinations | **10 415** | (100) | **1 614** | (15.5) |  |  |  |
|  |  |  |  |  |  |  |  |
| **Heavyweight mesh** | **1 206** | (12) | **221** | (18) |  |  |  |
| No fixation | 366 | (3.5) | 50 | (13.7) | **1** | *reference* | — |
| Tacks, absorbable | 343 | (3.3) | 67 | (20) | **1.5** | *0.96—2.2* | 0.077 |
| Tacks, metal | 343 | (3.3) | 73 | (21) | **1.7** | *1.1—2.5* | 0.016 |
| Fibrin glue | 154 | (1.5) | 31 | (20) | **1.6** | *0.99—2.7* | 0.056 |
|  |  |  |  |  |  |  |  |
| **Lightweight mesh** | **4 933** | (47) | **748** | (15) |  |  |  |
| No fixation | 1 554 | (15) | 265 | (17) | **1.3** | *0.95*—*1.8* | 0.093 |
| Tacks, absorbable | 990 | (10) | 155 | (16) | **1.2** | *0.86*—1.7 | 0.26 |
| Tacks, metal | 166 | (1.6) | 26 | (16) | **1.2** | *0.68*—*1.9* | 0.60 |
| Fibrin glue | 1 910 | (18) | 260 | (13.6) | **1.05** | *0.75*—*1.5* | 0.77 |
| Progrip | 313 | (3.0) | 42 | (13.4) | **0.97** | *0.62*—*1.5* | 0.88 |
|  |  |  |  |  |  |  |  |
| **3D mesh** | **3 894** | (37) | **600** | (15) |  |  |  |
| No fixation | 2 966 | (28) | 441 | (15) | **1.2** | *0.83*—*1.6* | 0.40 |
| Tacks, absorbable | 737 | (7.1) | 131 | (18) | **1.4** | *0.96*—*2.0* | 0.080 |
| Fibrin glue | 191 | (1.8) | 28 | (15) | **1.1** | *0.64*—*1.8* | 0.81 |
|  |  |  |  |  |  |  |  |
| **Uncategorized** combinations | |  |  |  |  |  |  |
| Rare or unspecified | **382** | (3.7) |  |  |  |  |  |
| Logistic regression, adjusted for the effect modifiers in Table 3.  Respondents %: denominator = all 10,525 patients.  CPIP %: n CPIP/n respondents, within each mesh–fixation category.  CPIP, chronic postoperative inguinal pain.  aOR, adjusted CPIP odds ratio. | | | | | | | |
